# Accidental Inclusions Following Blast Injury in Esthetical Zones: Ablation by a Hydrosurgery System

**Published:** 2012-07-26

**Authors:** Frank Siemers, Karl L. Mauss, Eirini Liodaki, Christian Ottomann, Philipp A. Bergmann, Peter Mailänder

**Affiliations:** Plastic Surgery, Hand Surgery, Burn Unit, University Hospital Schleswig-Holstein, Campus Lübeck, Ratzeburger Allee 160, 23538 Lübeck, Germany

## Abstract

In case of blast injuries, traumatic tattoos can result from accidental inclusions of intradermal pigmented particles. To avoid these tattoos, especially in esthetical areas like the head and neck region and the hands, the primary goal in our treatment is to remove all particles and foreign bodies. Superficial foreign bodies can easily be removed by brushes or dermabrasion. Deeper lesions are a challenge for plastic surgeons, because they are not so easily removed. Ablation by a water jet surgical tool, the Versajet-system (Smith & Nephew Inc, Andover, MA), enables the removal of nearly all particles and foreign bodies, which sustained a blast injury of the face or the hands. Aim of this paper is to describe the method of using this hydrosurgery system in blast injuries in esthetical zones and its advantages by presenting cases of two patients of us.

## PATIENTS AND METHODS

In 2009 and 2010, 7 patients with blast injuries in combination with accidental inclusions of intradermal pigmented particles were treated in our hospital. All patients were admitted within the first 4 hours following the injury. During admission on our burn unit, we performed a wound debridement and foreign body extirpation under general anesthesia. This procedure took 60 to 100 minutes depending on the number of foreign bodies. In contrast to our further treatment strategy when we removed the particles by dermabrasion, using a brush and physiological saline solution, we ablated the foreign bodies by use of the hydrosurgery tool Versajet.

**Figure 1:**
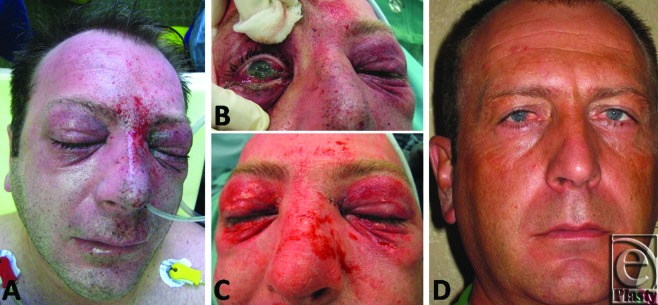
(*a*, *b*) Examination findings at admission to the hospital, with multiple foreign body tattoos within the eyes, eyelids, nose, and forehead. (*c*) After debridement with the Versajet hydrosurgery system. (*d*) Postoperative findings after 6 months.

**Figure 2:**
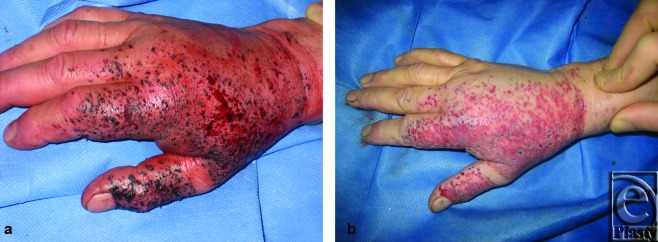
A 24-year-old patient underwent a blast injury by an explosion of firework with foreign bodies incorporation in his right hand. The use of the Versajet system allowed for a successful removal of foreign body particles with good functional and esthetic result.

In all cases, we used a standard hand-piece Versajet Plus, with a 45°-angled tip and a 14-mm working window. The operation was closed by the uptake of a dressing (Omiderm). The wounds healed in all cases with no complications at 2 weeks; 6 months postoperatively no significant scar formations are visible. To describe our method analytically, we would like to refer to the case of a 39-year-old male patient who had an industrial accident while welding. The explosion caused a blast injury with facial traumatic inclusion of foreign bodies. The patient was admitted 30 minutes following injury. During admission on our burn unit, physical examination revealed an irregular pattern of deeply embedded fine and rough particles 1 to 4 mm in diameter that affected the nose, cheeks, forehead, and both eyelids (Figs 1a and 1b). An injury of the medial corner of the left eye was treated surgically, whereas the Versajet hydrosurgery system was sufficiently used for wound debridement and foreign body extirpation under general anesthesia (Fig 1c). For the surgical procedure, we used a standard hand-piece with a 45°-angled tip and a 14-mm working window. Conjunctivas and lenses of both sides were cleaned under the operation microscope. The operation was closed by the uptake of a dressing (Omiderm, Omikron Scientific Limited, Rehovot, Israel). We would like also to present the case of a 24-year-old patient who underwent a blast injury by an explosion of firework with foreign bodies incorporation in his right hand (Fig 2a). The use of the Versajet system allowed for a successful removal of foreign body particles with good functional and esthetic result (Fig 2b).

## RESULTS

The wounds healed with no complications at 2 weeks; 6 months postoperatively, no significant scar formations are visible (Fig 1d). No infection of the wounds, no pain, no function or sensibility disorders were referred and the patients were very pleased with the esthetic result. Visual acuity is reduced about 20% in patients with foreign body incorporation in the eye.

## DISCUSSION

In case of accidental inclusions following blast injury, surgical debridement is necessary to avoid traumatic tattoos, especially in the most commonly affected, visible regions as head, neck, and hands.^1^^,^^2^ Best results are gained by immediate and complete removal of foreign-pigmented matters throughout the initial wound care. In general, superficial foreign bodies could easily removed by brushes. So far, persistent deeper particles which were removed by dermabrasion,^3^ surgical excision, cryosurgery, electrosurgery, or laser treatment. Despite these arrangements, visible scar formations can result.^4^^,^^5^

Deeply embedded pigmented particles that were not removed turn to traumatic tattoos. By use of Q-switched Nd:YAG short-pulse laser, a fragmentation of the particles without thermal damage to adjacent structures is achieved.^6^^-^8 Possible complications by use of this method were described by Fusade et al.^9^ In 3 cases, he observed hypertrophic scars and spreading of pigments in the skin around the initial tattoo following inclusions of gunpowder. Depending on his clinical experiences, Sunde et al^10^ stated that carbon dioxide laser may prove to be useful in the delayed treatment of traumatic tattoos. Cambier and Rogge^11^ achieved good and excellent results following removal of traumatic tattoos by use of an variable pulsed Erbium:YAG laser. In comparison with later dermabrasion, the laser procedure was more reliable and caused fewer adverse effects. El Sayed et al^12^ made good experiences by the use of a Q-switched ruby laser for the treatment of traumatic tattoos. Two conditions should be respected, the low fluency and the pretreatment test zone.

A new option for removal of traumatic inclusions in initial care after admission to a hospital is the use of the Versajet hydrosurgery device. This tool works by a high velocity water jet via an angled hand-piece, which allows removal of tissue in precise manner. The sterile saline stream runs through the opening of the hand-piece, tangential to the body surface. The pressure can be varied up to 10 steps. Especially in the lower settings, the Versajet system has a very good cleaning mechanism. Additional to the irrigation, the Venturi effect creates a localized vacuum that removes surface debris, which is sucked into the machine together with the irrigation fluid. The surgeon is able to prepare and cut injured tissue or foreign particles while aspirating debris by suction.

There is a use of water jet surgical tools in general surgical procedures,^13^^-^15 surgery of bone,^16^ or brain.^17^ Following further developments, techniques with the Venturi effect were established for the debridement of contaminated wound surfaces. First experiences in the use of the Versajet tool in burn surgery were published since 2005.^18^^-^20 The water jet allows a precise debridement of thermal damaged tissue. In comparison with conventional techniques with fixed settings, the Versajet system allows fine adjustments and protects vital tissue. Especially in contoured areas of the face, a precise debridement is possible by the use of a water-jet system. As an additional effect, Rennekampff et al^19^ describe a possible lower bacterial load in burn wounds. In comparison with dermabrasion, Cubison et al^18^ reported a lesser environmental contamination. The surgical procedure should close by the uptake of a biological dressing like Omiderm.^18^
